# The synergetic effect of bioactive molecule–loaded electrospun core‐shell fibres for reconstruction of critical‐sized calvarial bone defect—The effect of synergetic release on bone Formation

**DOI:** 10.1111/cpr.12796

**Published:** 2020-03-22

**Authors:** Zhenzhen Wu, Chongyun Bao, Shaobing Zhou, Tao Yang, Liao Wang, Mingzheng Li, Long Li, En Luo, Yingjie Yu, Yushu Wang, Xiaodong Guo, Xian Liu

**Affiliations:** ^1^ State Key Laboratory of Oral Diseases National Clinical Research Center for Oral Diseases West China Hospital of Stomatology Sichuan University Chengdu China; ^2^ Department of Periodontology and Implantology Stomatological Hospital Southern Medical University Guangzhou China; ^3^ Key Laboratory of Advanced Technologies of Materials Ministry of Education School of Materials Science and Engineering Southwest Jiaotong University Chengdu China; ^4^ Department of Biomedical Engineering Tufts University Medford MA USA

## Abstract

**Objectives:**

Bone regeneration is a complex process modulated by multiple growth factors and hormones during long regeneration period; thus, designing biomaterials with the capacity to deliver multiple bioactive molecules and obtain sustained release has gained an increasing popularity in recent years. This study is aimed to evaluate the effect of a novel core‐shell electrospun fibre loaded with dexamethasone (DEX) and bone morphogenetic protein‐2 (BMP‐2) on bone regeneration.

**Materials and methods:**

The core‐shell electrospun fibres were fabricated by coaxial electrospinning technology, which were composed of poly‐D, L‐lactide (PLA) shell and poly (ethylene glycol) (PEG) core embedded with BMP‐2 and DEX‐loaded micelles. Morphology, hydrophilicity, gradation, release profile of BMP‐2 and DEX, and cytological behaviour on bone marrow mesenchymal stem cells (BMSCs) were characterized. Furthermore, the effect on bone regeneration was evaluated *via* critical‐sized calvarial defect model.

**Results:**

The electrospun fibres were featured by the core‐shell fibrous architecture and a suitable degradation rate. The sustained release of DEX and BMP‐2 was up to 562 hours. The osteogenic gene expression and calcium deposition of BMSCs were significantly enhanced, indicating the osteoinduction capacity of electrospun fibres. This core‐shell fibre could accelerate repair of calvarial defects in vivo via synergistic effect.

**Conclusions:**

This core‐shell electrospun fibre loaded with DEX and BMP‐2 can act synergistically to enhance bone regeneration, which stands as a strong potential candidate for repairing bone defects.

## 1 INTRODUCTION

The repair of bone defects generated by trauma, tumour and congenital malformation remains a long‐standing challenge. It is reported that more than $2.5 billion is spent for over 2 million bone grafts in the United States each year.^1^ Considering the shortcoming of the limited bone donors of autografts and the potential risk of immune reactions of allografts,^2^, ^3^ the bone tissue engineering emerges as a promising alternative.^2^, ^4^ An essential issue in bone tissue engineering is to construct the biomaterials with biofunction to regulate cellular function such as the migration, proliferation and differentiation.^5^, ^6^, ^7^ However, conventional fabrication techniques, for example freeze‐drying,^8^ gas foaming^9^ and salt leaching,^10^ are incapable to fabricate the construct with various biofunction.^11^, ^12^

The bioactive molecule–loaded fibrous meshes fabricated through electrospinning technology, with fibre diameters ranging from several nanometres to a few micrometres and featured by an interconnected microporosity,^13^, ^14^, ^15^ have exhibited a great potential in bone tissue engineering.^16^ Nevertheless, traditional bioactive molecule–loaded electrospun fibres are faced with multi‐deficiencies. The bioactive molecule cargos exposed to organic solvents are prone to denature during electrospinning procedure.^5^ The lack of sustained release profile is another major shortcoming.^5^, ^17^ The core‐shell fibres fabricated by the coaxial electrospinning have emerged as a potent candidate to overcome aforementioned limitations. Attributed to the unique architecture, the bioactive molecules can be embedded into the polymer core under inorganic solutions, thus avoiding the potential denaturation. In addition, the burst release profile can be weakened to a great extent and the sustained release profile can be achieved via the shell structure. Moreover, through controlling the thickness and composition of the shell, the release rate of bioactive molecules can be further regulated.^12^, ^18^

The bone formation is a complex process involved plenty of growth factors and hormones in a spatiotemporal manner. Therefore, loading multiple bioactive molecules to the construct has been considered as an effective way to promote bone tissue regeneration. The bone morphogenetic protein‐2 (BMP‐2) characterized by its superior osteoinductive capacity has been widely used in bone tissue engineering.^19^, ^20^ The dexamethasone (DEX), a hydrophobic bioactive molecule commonly used in bone tissue engineering, is able to promote the osteogenic differentiation of mesenchymal stem cells (MSCs).^21^, ^22^ BMP‐2 and DEX improve cell proliferation and differentiation through different mechanisms. BMP‐2 can promote MSCs differentiation by the activation of downstream Smad pathways.^23^ DEX exerts its osteogenic activity via binding to the intracellular glucocorticoid receptor.^24^ Some studies also have demonstrated that the combinatory utilization of BMP‐2 and DEX exerted a synergistic effect on bone regeneration.^25^ Yoshikazu Mikami et al showed that DEX can enhance ALP expression with the presence of BMP‐2.^26^ However, owing to the lack of proper delivery carriers, employing high doses of BMP‐2 and DEX is inevitable which results in the high cost and undesirable side effects.^27^, ^28^, ^29^ To overcome the aforementioned shortcomings, fabricating dual‐bioactive molecule–loaded construct has been strongly advocated. Nevertheless, the current construct in bone tissue engineering is still far from perfect, as the release of bioactive molecule cannot endure during the bone regeneration which usually requires a few weeks or months.^30^ The superiorities of the core‐shell electrospun fibre, characterized by controllable release barriers (shell) and a sustainable release profile of bioactive molecules, are supposed to be a strong candidate to simultaneously load both BMP‐2 and DEX and achieve a long‐term sustainable release.^12^, ^18^

In this study, a dual‐bioactive molecule (BMP‐2 and DEX)–loaded core‐shell fibrous mesh was fabricated by the coaxial electrospinning technique. The shell consisted of poly‐D, L‐lactide (PLA), and the core was made up of poly (ethylene glycol) (PEG). Considering the hydrophobic nature of DEX, DEX was firstly encapsulated in micelles and then together with BMP‐2 was loaded inside the PEG core. The morphology of core‐shell fibrous mesh, degradation and release profile were investigated. The osteogenic differentiation and osteogenesis of this core‐shell fibrous mesh were also evaluated both in vitro and in vivo.

## 2 MATERIALS AND METHODS

### 2.1 Materials

mPEG, PEG (20 kDa), dexamethasone (DEX) and ε‐caprolactone (ε‐CL) were purchased from Sigma‐Aldrich Co. LLC. (MO, USA). PLA (Mw = 140 kDa) and mono‐methoxy poly (ethylene glycol)‐block‐poly (ε‐caprolactone) (mPEG‐PCL) were synthesized through ring‐opening polymerization of cyclic D, L‐lactide monomer as reported in the previous literature.^31^ Recombination human BMP‐2 was purchased from Bioss Biological Technology Co., LTD. ε‐CL was dried and distilled before using. All other chemicals and solvents were purchased from Chengdu Kelong Chemical Reagents and used without further purification.

### 2.2 Preparation of the DEX‐loaded micelles

The blank and DEX‐loaded micelles were fabricated by a solvent evaporation method.^32^ Briefly, 0.1 g of mPEG‐PCL copolymer was dissolved in 10 mL tetrahydrofuran (THF) prior to dissolving 100 μg of DEX. Then, the copolymer solution was added dropwise into 10 mL deionized water under violent stirring. The mixed solution was moderately stirred for 4 hours at room temperature to completely evaporate the THF and thereby forming polymeric micelle solution. Subsequently, the DEX‐loaded micelle solution was dialysed against the deionized water by a dialysis bag (MWCO 1000) to remove the unloaded DEX before freeze dried to obtain the micelle powder. Measured by UV‐vis spectrophotometer (UV‐2550, Shimadzu, Japan), DEX loading content (LC) was 45.7 ± 1.1% and encapsulation efficiency (EE) was 84.1 ± 3.4%.

### 2.3 Coaxial electrospinning

The coaxial electrospinning process was conducted following the previous work.^33^ PLA was dissolved in a mixed solvent of chloroform (CF) and dimethylformamide (DMF) (v/v was 1:3), forming a 14% w/v electrospinning shell solution. As for constructing different fibrous meshes, four types of electrospinning core solution were prepared by adding different components into 10% w/v PEG/deionized water, including pure PEG solution (the blank group), 0.45 g of mPEG‐PCL micelles in PEG solution (PLA/PEG‐DEX), 5 μg of BMP‐2 in PEG solution (PLA/PEG‐BMP‐2), 5 μg of BMP‐2 and 0.45 g of mPEG‐PCL micelles in PEG solution (PLA/PEG‐DEX‐BMP‐2). A coaxial spinneret connected to a high voltage supply was used to fabricate core‐shell fibrous meshes. The flow rates of the shell solution and the core solution were controlled at 1.5 mL/h and 0.6 mL/h, respectively. The volume of the core solution was 6 mL, and the volume of the shell solution was 15 mL. The distance between the spinneret and the collector was kept at 17 cm. The fibres were collected on an aluminium collector under an applied voltage of 22 ± 0.5 kV. All the collected fibres were dried in a vacuum at room temperature for over 1 week.

### 2.4 Characterization of different core‐shell fibres

The morphologies of different fibres were characterized by scanning electron microscopy (SEM) (Quanta 200, Philips). The fibre diameter was measured from the SEM images using ImageJ software. Transmission electron microscopy (TEM) (JEM‐2100F, JEOL) was used to verify the core‐shell structures of different fibre samples. The water contact angles were measured by contact angle goniometer (Model JY‐82, Chengde Experimental Machine Plant).

### 2.5 Degradation test

The degradation of the electrospun fibres was performed in phosphate‐buffered saline (PBS) at 37°C. The dried fibrous meshes were accurately weighed (20 mg) and then immersed in 25 mL PBS in test tubes. The tubes were kept in a thermostated shaking air bath maintained at 37°C and 120 cycles/min. The samples were taken at 4, 8 and 12 weeks, rinsed with deionized water to remove residual buffer salts, and dried in vacuum. The degradation percentage was determined by dividing the dry weight remained by initial weight. The molecular weight was determined using gel permeation chromatography (GPC, Waters) analysis.

### 2.6 In vitro drug release

To evaluate the drug release of PLA/PEG‐DEX, PLA/PEG‐BMP‐2 and PLA/PEG‐DEX‐BMP‐2 fibres, three samples (n = 3) weighing about 6 mg of each group were immersed in tubes containing 2 mL PBS. At the predetermined time, the release medium in the tubes was carefully collected and replaced by fresh PBS. The concentration of DEX in the release medium was tested using ultraviolet spectrophotometer at 242 nm,^34^ and BMP‐2 was measured by a BMP‐2 ELISA kit (Boster Biological Engineering Co., Ltd).

### 2.7 Proliferation and morphology of BMSCs on core‐shell fibrous meshes

#### 2.7.1 Cell culture and seeding on fibrous meshes

The bone mesenchymal stem cells (BMSCs) were harvested from 2‐week‐old SD rats using the whole bone marrow culture method. The BMSCs were cultured in Dulbecco's modified Eagle's medium (DMEM) supplemented with 10% foetal bovine serum (FBS) and 1% penicillin/streptomycin. Cells at passage 3‐4 were used in this study. All the electrospun fibrous meshes were sterilized with an ultraviolet lamp for 2 hours on each side. 2 × 10^4^ cells were seeded on the surfaces of fibrous meshes in 24‐well plates. The cells were cultured in osteogenic culture medium (DMEM supplemented with 50 μg/mL ascorbic acid and 10 mmol/L glycerol phosphate), which was refreshed every 2 days.

#### 2.7.2 Cell viability and phenotypes observation

After culturing for 4, 7 and 14 days, the viability of cells on fibrous meshes was investigated by the Live/Dead Viability/Cytotoxicity kit (Molecular Probes, USA). The percentage of live cells was measured as follows: L = number of live cells/ (number of live cells + number of dead cells) × 100%. The live cell density was measured as follows: D = number of live cells in the image/ the image area.^35^, ^36^ Views of three random fields were observed for each sample. Five samples of each group yielded 15 images at each time point.

The immunocytochemical staining technique was employed to further observe the morphology of cells on fibrous meshes. After 7 days, the samples were fixed with 4% paraformaldehyde and washed with PBS. Then, Alexa Fluor 488 phalloidin and DAPI (Invitrogen) were added to stain F‐actin and nuclei. The samples were observed with the epifluorescence microscope (TE2000S, Nikon).

### 2.8 Osteogenic differentiation of BMSCs

#### 2.8.1 Alizarin red staining

Alizarin red staining was used to evaluate the extent of mineral deposition of BMSCs. The samples seeded with BMSCs were fixed with 4% paraformaldehyde and stained with 0.1% alizarin red after 7 and 14 days of culture.

#### 2.8.2 Osteogenic gene expression

The expression of the osteogenic differentiation marker genes of BMSCs was evaluated by real‐time quantitative polymerase chain reaction (qPCR). After 7, 14 and 21 days, the total RNA was extracted using TRIzol reagent according to the manufacturer's instruction. Then, the RNA was reversely transcribed into cDNA with a PrimeScript RT Reagent Kit. qPCR was carried out with SYBR Premix Ex Taq. Alkaline phosphatase (ALP), osteocalcin (OCN) and osteopontin (OPN) gene were selected as target gene, and glyceraldehyde 3‐phosphate dehydrogenase (GAPDH) was used as a control.

### 2.9 In vivo animal experiment and surgical procedures

All animal care and experiments were conducted under the supervision of the Animal Research Committee of the West China School of Stomatology, Sichuan University (WCHSIRB‐D‐2016‐150). Twenty female Sprague‐Dawley rats were randomly divided into 4 groups (n = 5). The rats were anaesthetized with 10% chloral hydrate (4 mL/kg). A mid‐longitudinal incision was made over the calvarium, and full‐thickness flaps were elevated. An 8 mm circular bone defect was created in the calvarium with a trephine bur. The cranial defect was covered with PLA/PEG fibrous meshes, PLA/PEG‐DEX fibrous meshes, PLA/PEG‐BMP‐2 fibrous meshes or PLA/PEG‐DEX‐BMP‐2 fibrous meshes. The wound was then carefully sutured.

### 2.10 Analysis of new bone formation

After 12 weeks of implantation, the rats were sacrificed and the whole calvarium was collected and fixed in 4% paraformaldehyde solution for 24 hours. Then, the specimens were prepared for micro‐computed tomography (micro‐CT) analysis and histology observation.

Following fixation, the specimens were scanned by a micro‐CT (ZKKS‐MCT‐Sharp, China). The defect region was identified by a cylindrical region of interest (ROI), and then, the bone mineral density (BMD) and bone volume/tissue volume (BV/TV) were calculated for quantitative analysis of bone formation within this region.

After micro‐CT scanning, the specimens were decalcified with EDTA, dehydrated through a series of ethanol solutions and embedded in paraffin. The central regions of circular defects were cut into thick sections of 5 μm. The sections were stained with HE as well as Masson's trichrome and observed through light microscopy (BX51, Olympus).

### 2.11 Statistical analysis

The data were described as the mean ± standard deviation. One‐way ANOVA followed by the LSD test was used to assess statistical significance. The level of significance was set as *P* < .05.

## 3 RESULTS

### 3.1 Characterization of different core‐shell fibres

**Figure 1:**
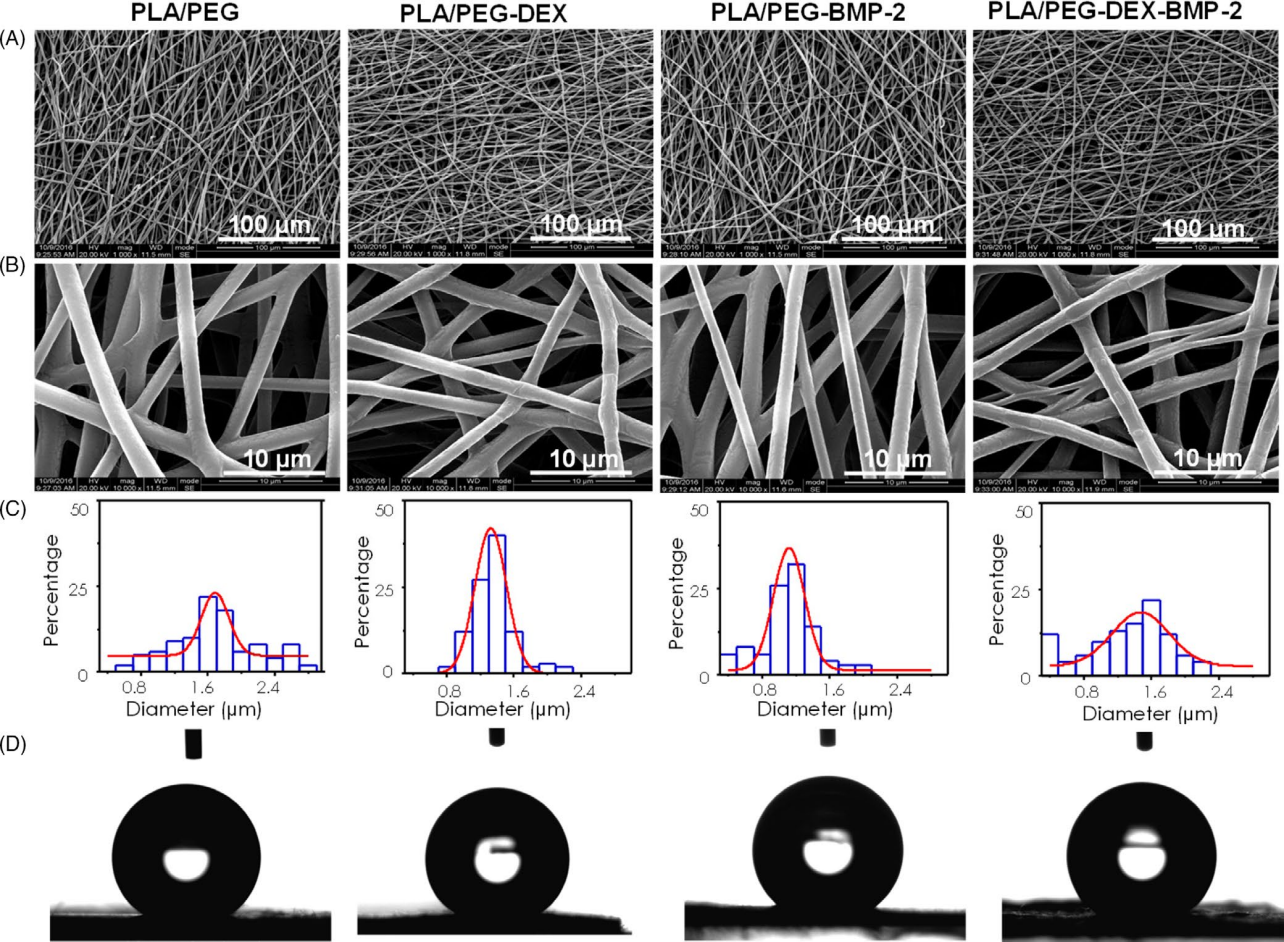
SEM images, histograms of corresponding diameter distributions and water contact angle of electrospun fibres

**Figure 2:**
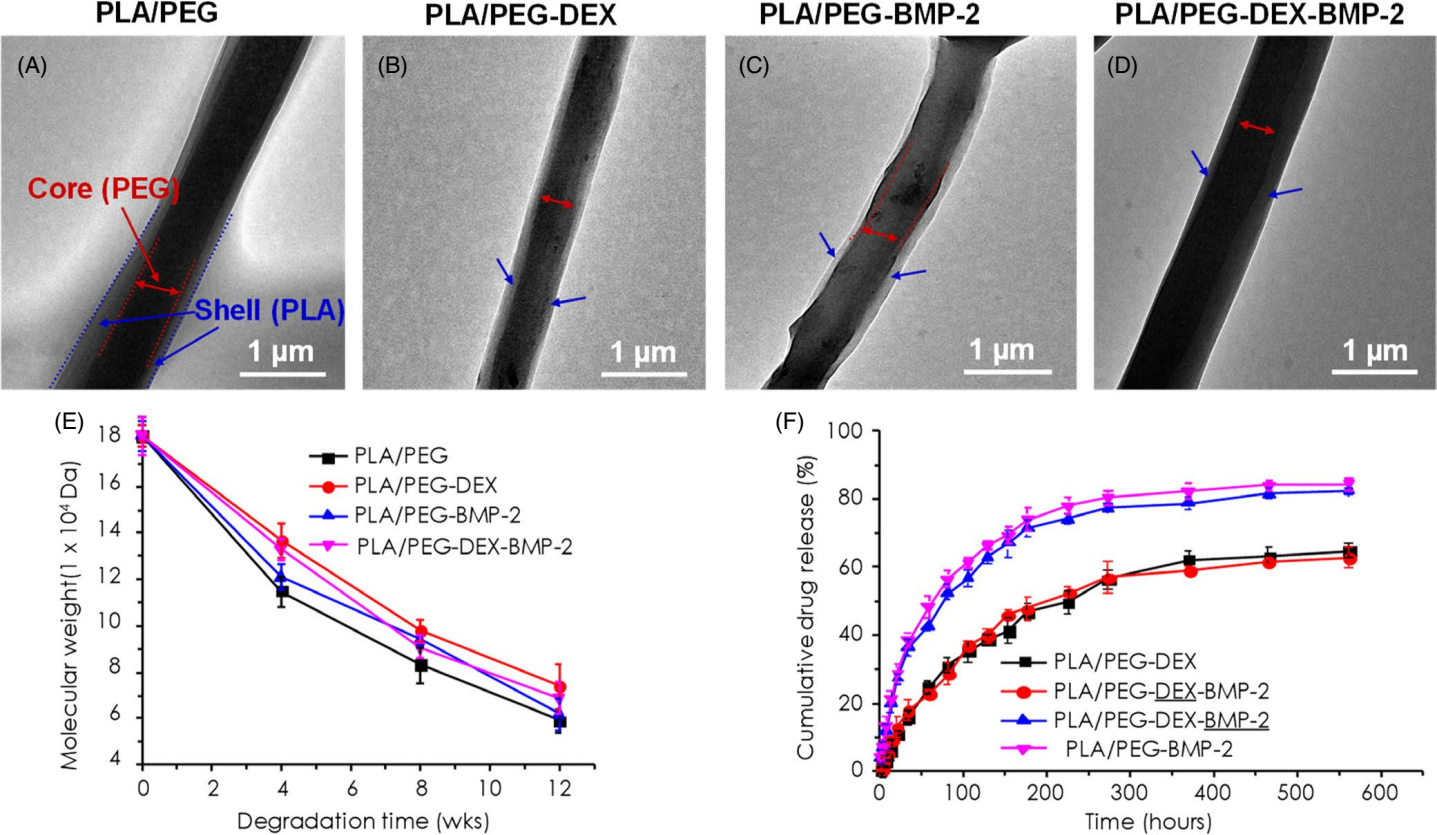
(A‐D) TEM images, (E) the degradation test result and (F) in vitro cumulative drug release profiles of electrospun fibres. Black and red represent the release profiles of DEX from PLA/PEG‐DEX and PLA/PEG‐DEX‐BMP‐2 fibres, while blue and pink represent the release profiles of BMP‐2 from PLA/PEG‐DEX‐BMP‐2 and PLA/PEG‐BMP‐2 fibres. The bars in E and F indicated the standard deviation

The microstructure of fibrous meshes was investigated by SEM. All the fibrous meshes consisted of interconnected and continuous fibres (Figure 1A,B), whose diameter was almost comparable among them. Analysed by ImageJ software, the average diameters of the PLA/PEG‐DEX, PLA/PEG‐BMP‐2 and PLA/PEG‐DEX‐BMP‐2 fibres were 1.35 ± 0.26 μm, 1.12 ± 0.34 μm and 1.31 ± 0.49 μm, respectively, which exhibited no significant difference with that of the PLA/PEG fibres (1.69 ± 0.51 μm) (Figure 1C). The data indirectly demonstrated that the encapsulated BMP‐2 and DEX did not affect the architecture of electrospun fibres. The water contact angle of PLA/PEG, PLA/PEG‐DEX, PLA/PEG‐BMP‐2 and PLA/PEG‐DEX‐BMP‐2 was approximately 139.98°± 2.22, 140.00°± 2.37, 136.08°± 4.48 and 135.90°± 4.33, respectively (Figure 1D). Meanwhile, the microstructure of fibrous meshes was also tested by TEM (Figure 2A‐D). The PLA/PEG fibres exhibited a high‐contrast core and a low‐contrast shell. This typical core‐shell structure was presented in all bioactive molecule–loaded fibres, which indicated that embedding DEX and BMP‐2 could not affect the core‐shell structure.

### 3.2 In vitro degradation test and bioactive molecules release

The change in molecular weight of electrospun fibrous meshes during degradation test was shown in Figure 2E. The molecular weight of all fibres gradually decreased. Generally, the PLA/PEG fibrous meshes exhibited the highest degradation rate in comparison with the bioactive molecule–loaded fibrous meshes, and the PLA/PEG‐DEX fibres revealed the lowest degradation rate.

The release profiles of BMP‐2 and/or DEX in different fibres were presented in Figure 2F. A biphasic release profile was observed for all the bioactive molecule–loaded fibrous meshes, which was characterized by a short burst release phase and a relatively long sustained release phase. For the BMP‐2, approximately 28.82% and 27.02% of cargo was, respectively, released from PLA/PEG‐BMP‐2 and PLA/PEG‐DEX‐BMP‐2 fibrous meshes after the initial burst release (around 22 hours). Subsequently, a sustained release emerged, which continued for more than 274 h. As for DEX, a rather weak initial burst (around 22 hours) of the DEX was detected, approximating 10.85% from PLA/PEG‐DEX and 12.99% from PLA/PEG‐DEX‐BMP‐2 fibrous meshes. The sustained release of DEX lasted for more than 466 hours. At the end of the release study (562 hours), the accumulative release amount of BMP‐2 reached almost 82.24% in comparison with 62.62% of DEX in PLA/PEG‐DEX‐BMP‐2 fibrous meshes.

### 3.3 Proliferation and morphology of BMSCs on core‐shell fibrous meshes

**Figure 3:**
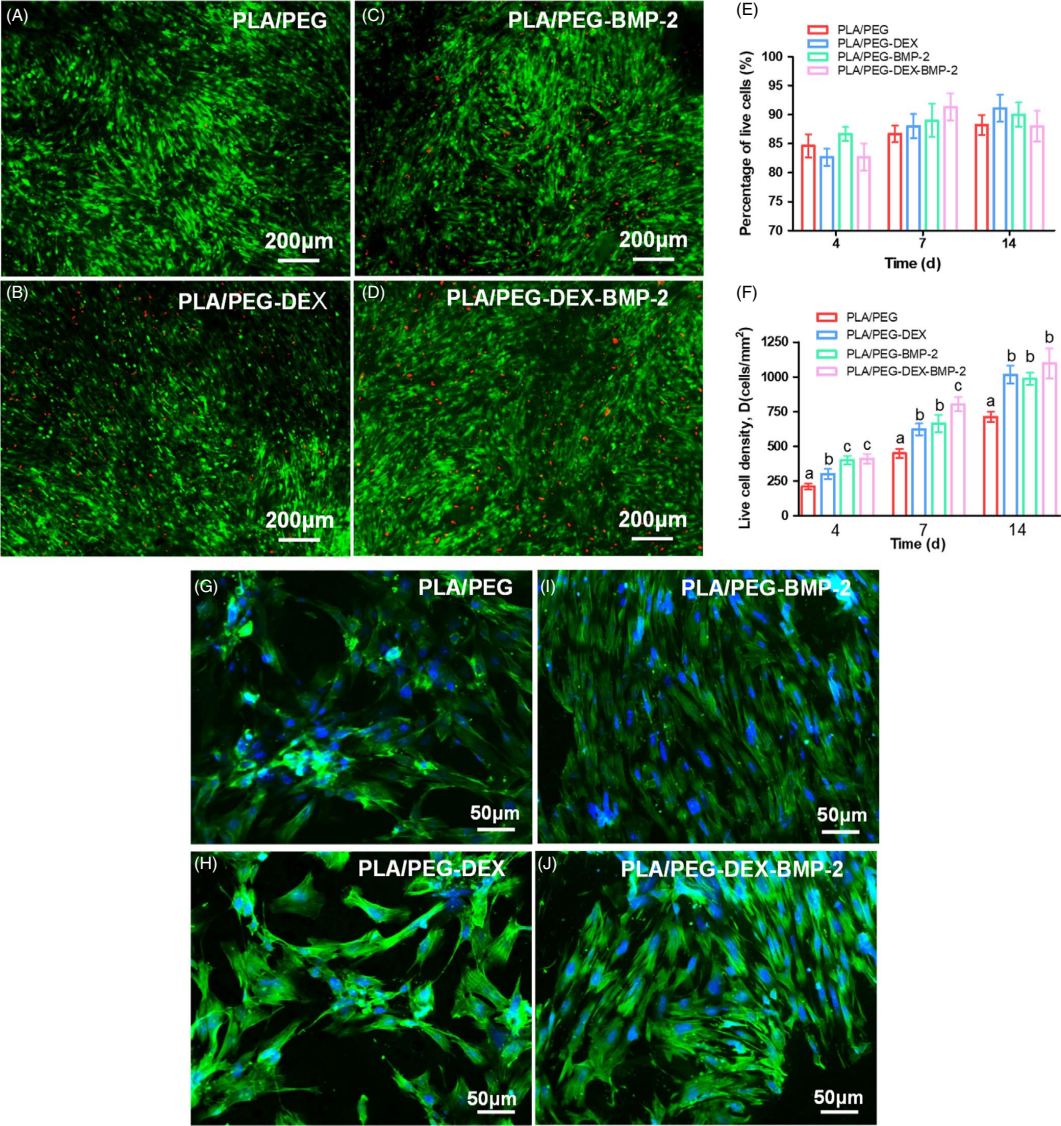
Representative live/dead images of BMSCs on fibres cultured at 14 d: (A) PLA/PEG, (B) PLA/PEG‐DEX, (C) PLA/PEG‐BMP‐2 and (D) PLA/PEG‐DEX‐BMP‐2. Among these, the live cells were stained green, while dead cells were stained red. (E) Percentages of live cells and (F) live cell density at 4, 7 and 14 d. At each time point, there was no significant difference among the data marked by the same letter, and significant difference existed among group marked by different letters (*P* < .05, n = 5, using ANOVA analysis, the bars indicated the standard deviation). The representative images of BMSCs stained by F‐actin at 7 d of (G) PLA/PEG, (H) PLA/PEG‐DEX, (I) PLA/PEG‐BMP‐2 and (J) PLA/PEG‐DEX‐BMP‐2

The live/dead images of BMSCs seeded on fibrous meshes were displayed in Figure 3A‐D. The overwhelming majority of cells was alive (stained green) and well‐spread, and only a few dead cells (stained red) were detected, indicating the excellent cytocompatibility of all the fibrous meshes. The number of live cells accounted for approximately 80%‐90% of all the cells (Figure 3E).

The live cell density increased over time in all fibrous meshes due to cell proliferation (Figure 3F). The density of live cells of bioactive molecule–loaded fibrous meshes (PLA/PEG‐DEX, PLA/PEG‐BMP‐2 and PLA/PEG‐DEX‐BMP‐2) was significantly increased compared with the PLA/PEG fibrous meshes at 4, 7 and 14 days. At 4 days, the cell proliferation of BMP‐2–loaded fibrous meshes (PLA/PEG‐BMP‐2 and PLA/PEG‐DEX‐BMP‐2) was significantly higher than that of PLA/PEG‐DEX fibrous meshes. At 7 days, significant difference in cells proliferation existed between the dual‐bioactive molecule–loaded fibrous meshes and the single bioactive molecule–loaded fibrous meshes. However, there was no significant difference in proliferation among the bioactive molecule–loaded fibrous meshes at 14 days. At 7 days, BMSCs adhered on all the scaffolds and formed interconnected multicellular networks (Figure 3G‐J).

### 3.4 Alizarin red staining

**Figure 4:**
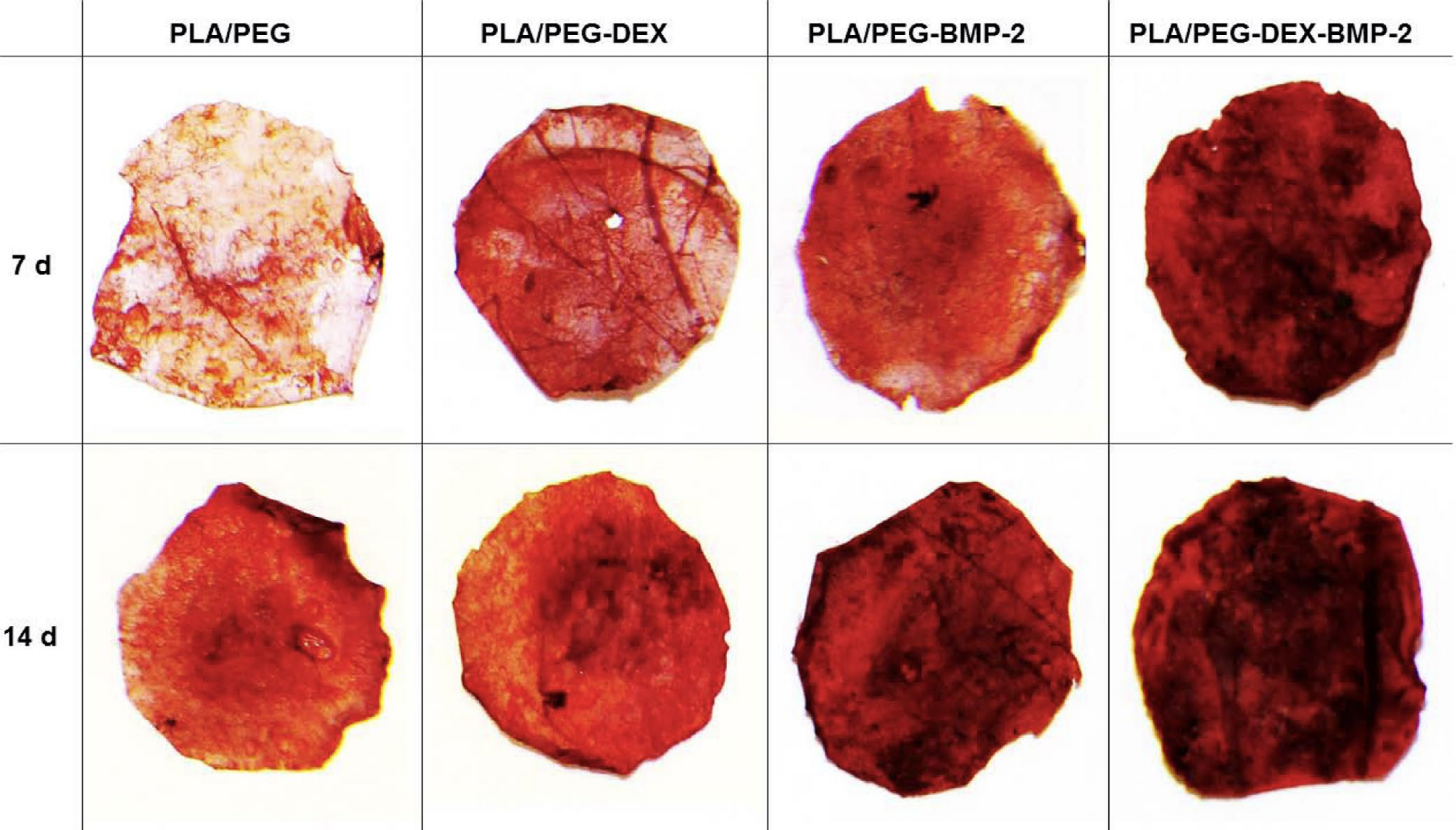
Alizarin red staining after BMSCs seeded on four fibres at 7 and 14 d

The calcium depositions of BMSCs on the fibrous meshes were investigated by the alizarin red staining at 7 and 14 days (Figure 4). Alizarin red staining displayed a time‐ and fibre‐dependent manner. At 7 days, contrary to the negative staining of the control fibrous meshes, all the bioactive molecule–loaded fibrous meshes exhibited positive staining, among which the dual‐bioactive molecule–loaded fibrous meshes displayed the maximum staining intensity. At 14 days, a weak positive staining emerged in control fibrous mesh, and staining intensity of the bioactive molecule–loaded fibrous meshes was further enhanced. The staining intensities were ranked in the following order: PLA/PEG‐DEX‐BMP‐2 fibrous mesh >PLA/PEG‐BMP‐2 fibrous mesh >PLA/PEG‐DEX fibrous mesh.

### 3.5 Osteogenic genes expression

**Figure 5:**
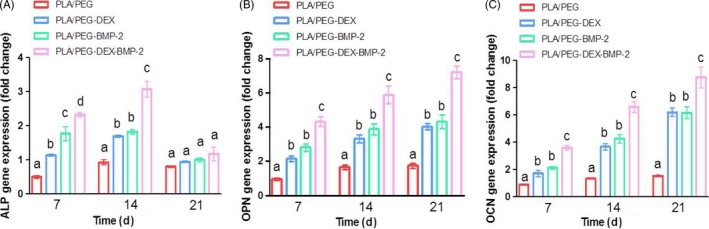
(A) ALP, (B) OPN and (C) OCN gene expressions in BMSCs seed on four scaffolds for 7, 14 and 21 d using qPCR. At each time point, there was no significant difference among the data marked by the same letter, and significant difference existed among group marked by different letters (*P* < .05, n = 3, using ANOVA analysis, the bars indicated the standard deviation)

The expression of osteogenic genes, including ALP, OCN and OPN, was determined by qPCR (Figure 5). As an early differentiation marker of BMSCs, ALP was moderately expressed at 7 days, reached the maximum expression at 14 days, and then displayed a decreasing expression at 21 days in all the fibrous meshes. OCN and OPN, which was expressed in the middle to late stage of differentiation, displayed an increasing expression level during culture and approached the expression peak at 21 days.

Besides the culture time, the gene expression was closely related to the bioactive molecules. At 7 and 14 days, the expression of ALP of the bioactive molecule–loaded fibrous meshes was significantly higher than that of the control fibrous meshes. And among them, BMSCs cultured on the dual‐bioactive molecule–loaded fibrous meshes exhibited a significantly higher ALP expression level than that on the single bioactive molecule–loaded fibrous meshes. Similarly, the OCN and OPN expression showed a comparable trend at 7, 14 and 21 days.

### 3.6 Micro‐CT

**Figure 6:**
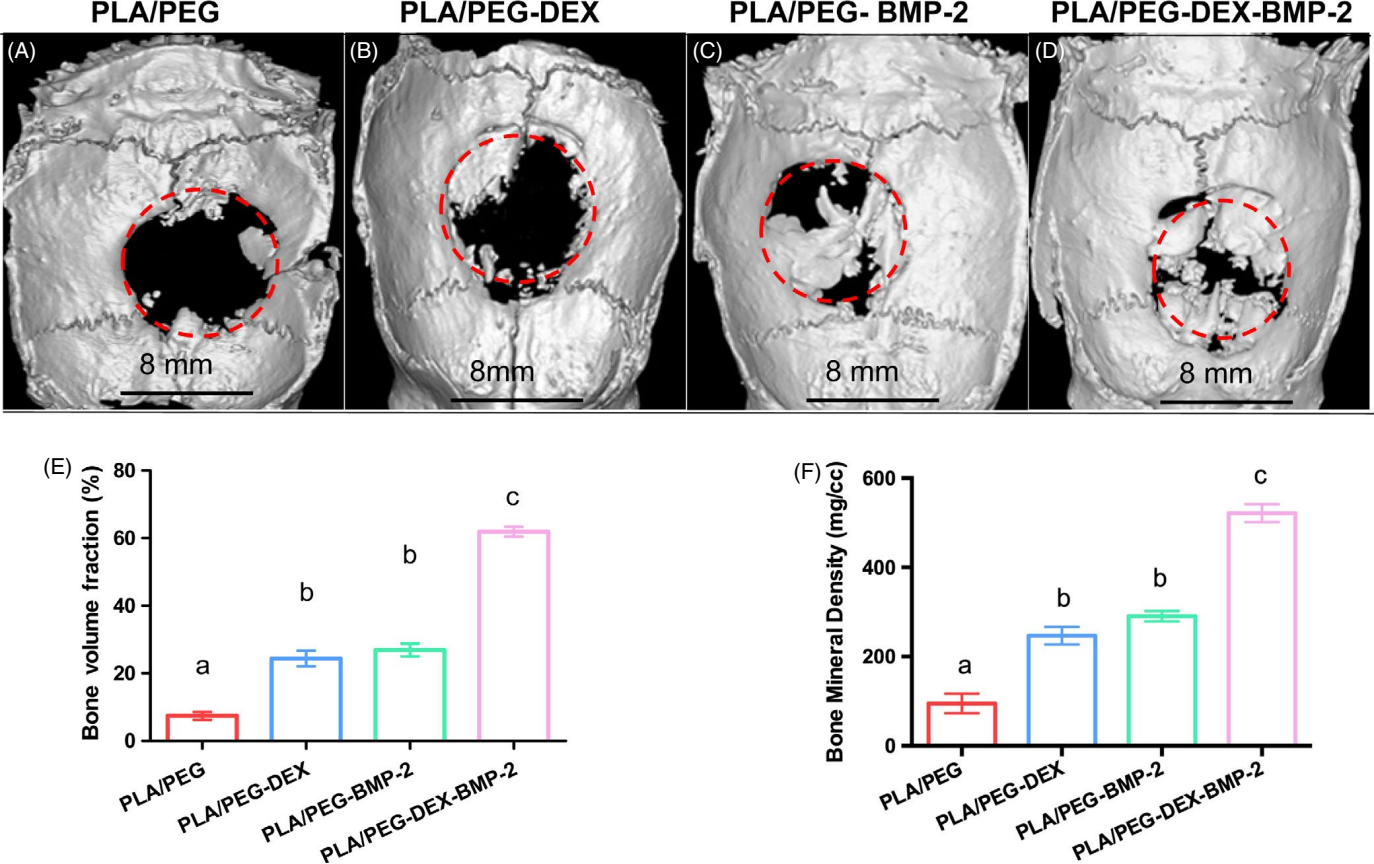
The micro‐CT images of (A) PLA/PEG, (B) PLA/PEG‐DEX, (C) PLA/PEG‐BMP‐2 and (D) PLA/PEG‐DEX‐BMP‐2 after implantation in vivo for 12 wk, the analysis of (E) new bone volume fraction and (F) bone mineral density of four groups. There was no significant difference among the data marked by the same letter, and significant difference existed among group marked by different letters (*P* < .05, n = 5, using ANOVA analysis, the bars indicated the standard deviation)

The repair of bone defect of different fibrous meshes was assessed in critical‐sized calvarial defect rat model. The 3D reconstructed images were presented in Figure 6A‐D. For the PLA/PEG group, there was almost no hard tissue formation in the bone defect region. A moderate amount of new bone formation was observed in the defect regions of PLA/PEG‐BMP‐2 and PLA/PEG‐DEX groups. The majority of defect in PLA/PEG‐DEX‐BMP‐2 group was filled with new bone. The quantification analysis of bone volume fraction was presented in Figure 6E. The bone volume fraction of PLA/PEG‐DEX‐BMP‐2 group reached 61.90 ± 2.08%, which was significantly higher than that of PLA/PEG‐DEX and PLA/PEG‐BMP‐2 groups (24.38 ± 3.23% and 26.92 ± 2.70%, respectively). The bone volume fraction of control group was only 7.40 ± 1.63% and was significantly lower than that of the bioactive molecule–loaded fibrous meshes. Similarly, the BMD of PLA/PEG group was 94.8 ± 22.0 mg/cc (Figure 6F). The BMD increased to 247.1 ± 19.7 mg/cc in PLA/PEG‐DEX group and 290.6 ± 11.8 mg/cc in PLA/PEG‐BMP‐2 group. The BMD significantly grew to 521.7 ± 20.1 mg/cc in the PLA/PEG‐DEX‐BMP‐2 group.

### 3.7 HE and Masson's trichrome staining

**Figure 7:**
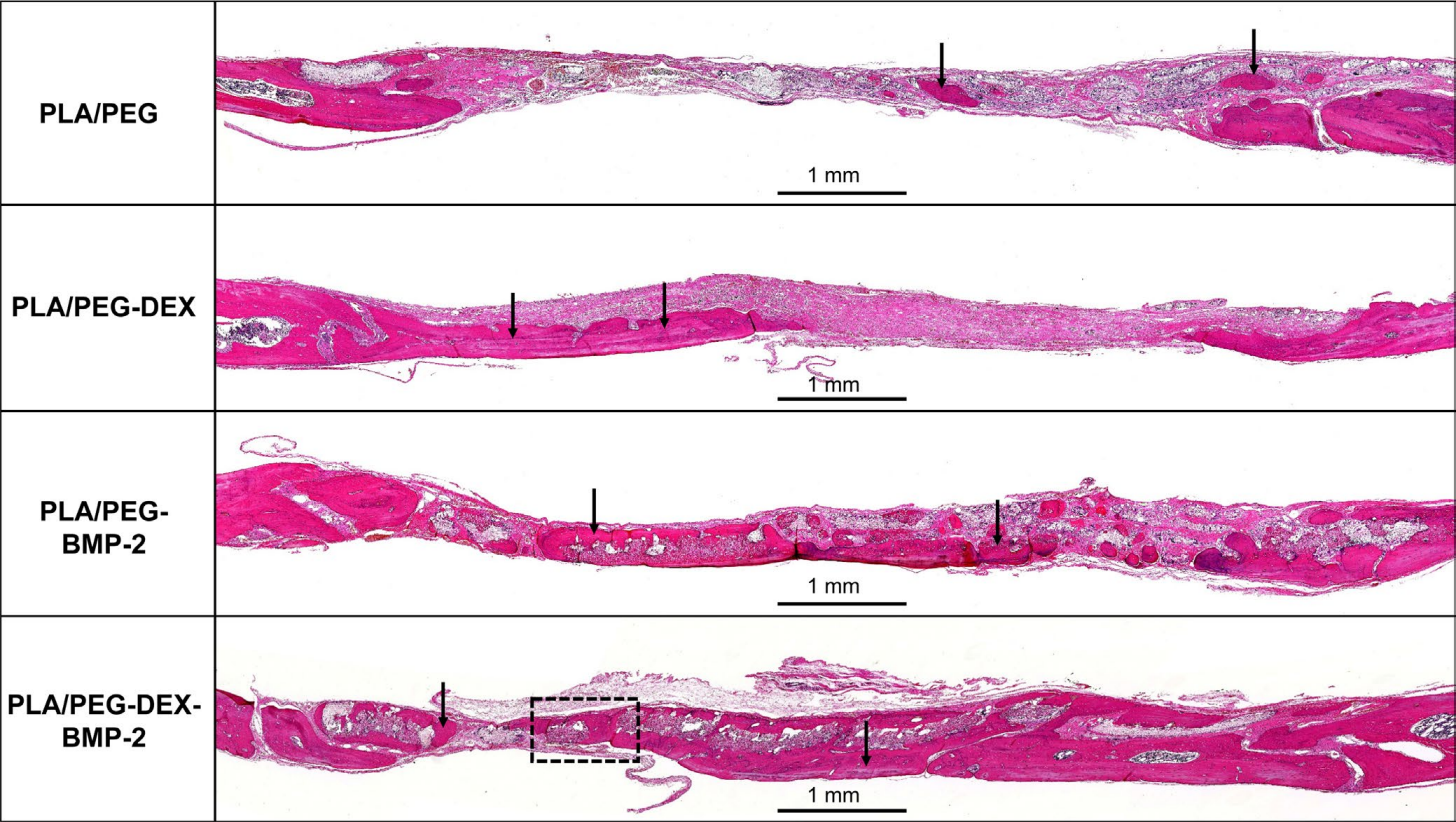
HE staining of PLA/PEG, PLA/PEG‐DEX, PLA/PEG‐BMP‐2 and PLA/PEG‐DEX‐BMP‐2 after implantation in vivo for 12 wk. The black arrows indicate areas of new bone

**Figure 8:**
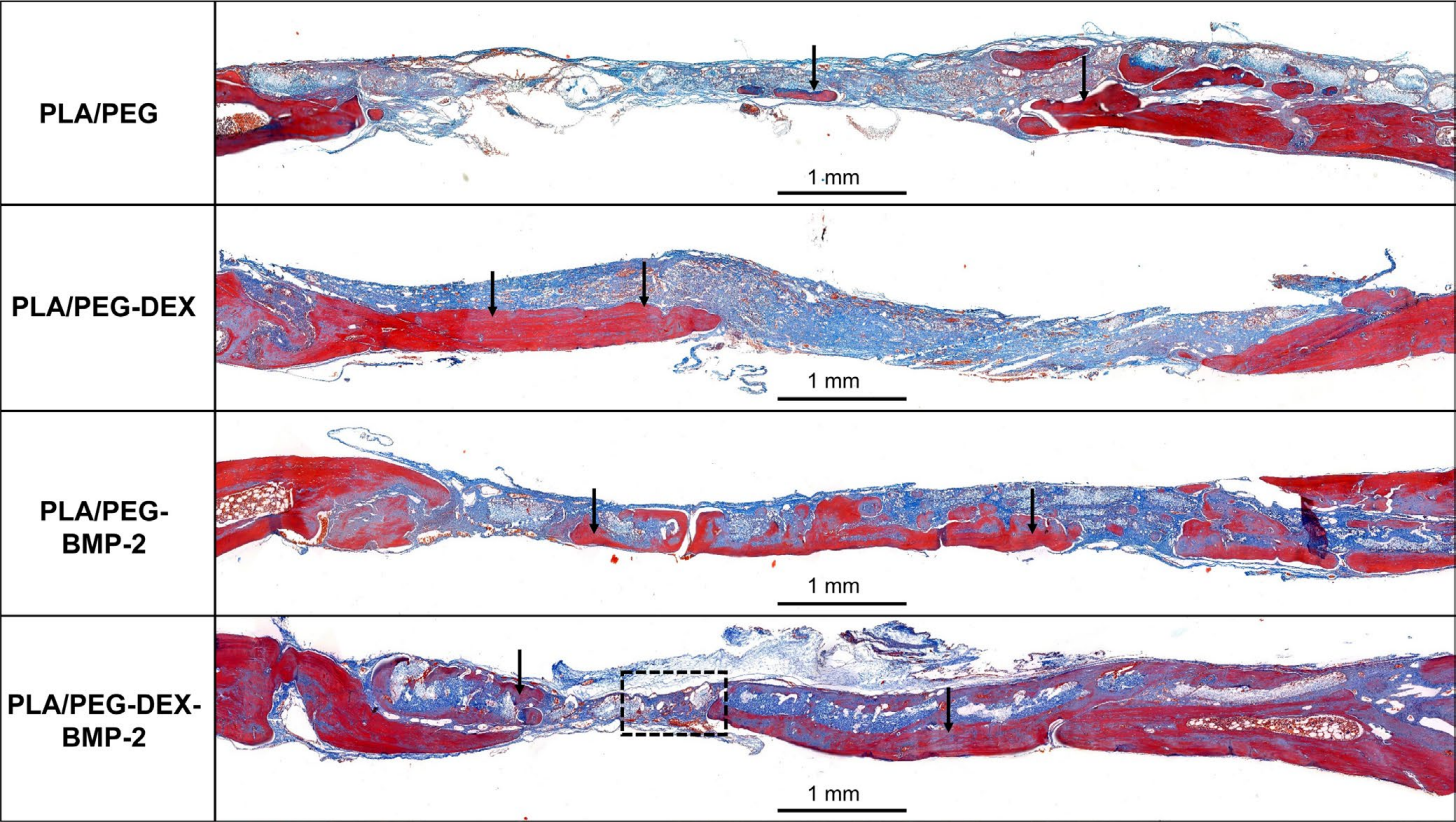
Masson's trichrome staining of PLA/PEG, PLA/PEG‐DEX, PLA/PEG‐BMP‐2 and PLA/PEG‐DEX‐BMP‐2 after implantation in vivo for 12 wk. The black arrows indicate areas of new bone

**Figure 9:**
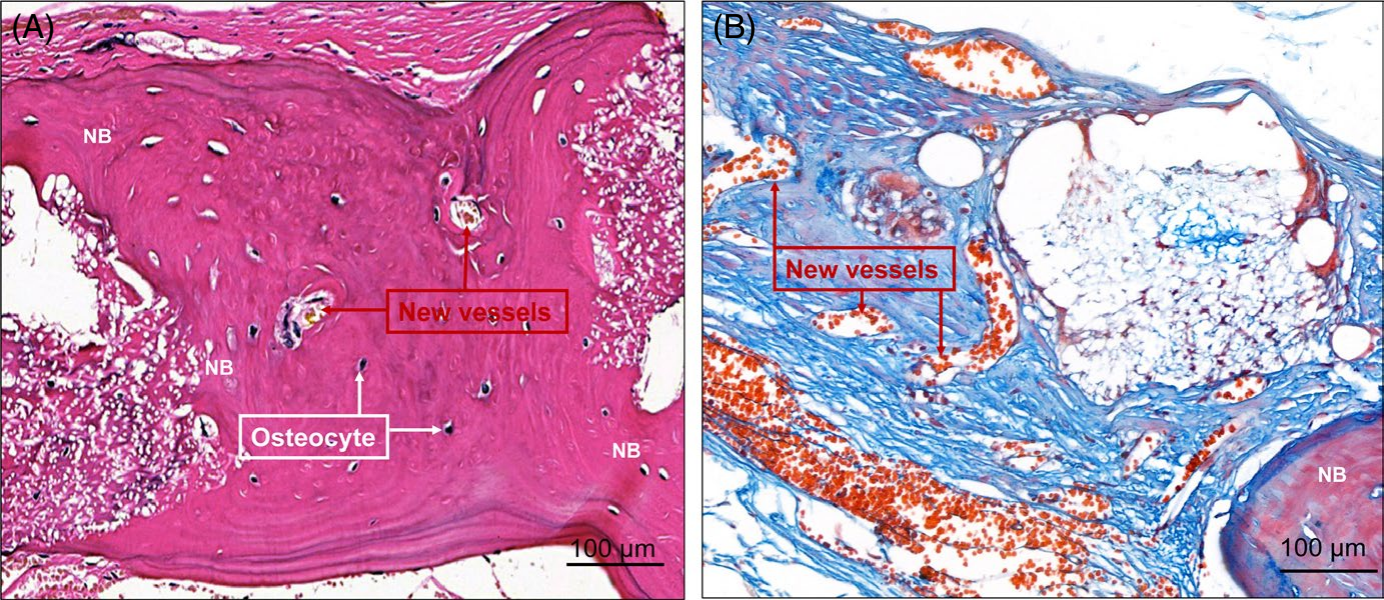
High magnification of (A) HE and (B) Masson's trichrome staining of PLA/PEG‐DEX‐BMP‐2 after implantation in vivo for 12 wk

HE and Masson's trichrome staining were performed to further evaluate the new bone formation. As shown in Figures 7 and 8, extremely limited bone regeneration was detected in the control group, and the defect regions were filled with extensive fibrous connective tissue. In the PLA/PEG‐DEX and PLA/PEG‐BMP‐2 groups, a moderate amount of new bone was formed in defect regions. The PLA/PEG‐DEX‐BMP‐2 group presented massive bone formation. At 12 weeks, the majority of fibrous meshes were degraded. Notably, new bone was mainly formed at the centres and the boundaries of defects in the PLA/PEG‐BMP‐2 group and PLA/PEG‐BMP‐2 group, whereas new bone was mainly formed at the boundaries in the PLA/PEG‐DEX group. Figure 9 showed the high magnification of HE and Masson's trichrome staining of PLA/PEG‐DEX‐BMP‐2 group. New bone was formed in defect regions, and some new blood vessels could be observed.

## 4 DISCUSSION

The bone regeneration is a complex process modulated by multiple growth factors and hormones.^37^, ^38^ Therefore, engineered constructs loaded with multiple and therapeutically relevant molecules are considered to be the potent candidates in promoting bone regeneration.^39^, ^40^ Although many studies have employed the electrospun fibrous meshes to load the bioactive molecule to promote the bone regeneration, most of them only loaded a single kind of biomolecule. Therefore, an electrospun fibrous mesh with the capacity to load the BMP‐2 and DEX was developed in this study. To obtain the sustained release of both biomolecules, a core‐shell fibrous mesh containing biomolecules was fabricated via coaxial electrospinning and employed to exert a synergistic effect during osteogenesis.

To control the sustained release of the two bioactive molecules, DEX‐loaded micelles and BMP‐2 were incorporated into the core. The release of bioactive molecules involved two mechanisms: the passive diffusion and the degradation of the polymers which was donated as the erosion‐coupled mechanism.^30^ Initially, the bioactive molecules were released from fibres due to passive diffusion. With the proceeding of polymer degradation, the passive diffusion‐driven release gradually changed into the diffusion/erosion‐coupled release. Notably, BMP‐2 was released faster than DEX in the initial stage. Since the DEX was encapsulated inside the micelles, there might be two paths for its release from fibres before polymer degradation. One was that DEX‐loaded micelles were first released from the fibre shell prior to DEX release; the other one was that DEX successively passed through the micelles and the shell in sequence. As for BMP‐2, it could directly efflux through the shell owing to the lacking of micelles. Therefore, BMP‐2 exhibited a faster release rate than DEX.

Proliferation, maturation and mineralization of extracellular matrix are three major stages during the growth and differentiation of MSCs.^41^ The enhancement of cell proliferation in PLA/PEG‐DEX, PLA/PEG‐BMP‐2 and PLA/PEG‐DEX‐BMP‐2 fibrous meshes was attributed to the encapsulated bioactive molecules, which have been reported to stimulate cellular proliferation.^42^, ^43^ The enhanced proliferation also indirectly indicated that the bioactivity of DEX and BMP‐2 maintained during the coaxial electrospinning process. Due to more BMP‐2 was released from fibrous mesh than DEX at initial stage, the cell proliferation was higher in PLA/PEG‐BMP‐2 group than PLA/PEG‐DEX at 4 days. At 7 days, the dual‐bioactive molecule–loaded fibrous meshes exhibited the highest cell proliferation, which might be associated with the synergistic effect of BMP‐2 and DEX. However, at 14 days, there was no significant difference in cell proliferation among bioactive molecule–loaded fibrous meshes. This might be related to the differentiation of MSCs induced by BMP‐2 and DEX, and thus slowing down their proliferation.^44^

The mechanisms of osteogenic differentiation induced by DEX and BMP‐2 are distinct. BMP‐2, a member of TGF‐β, binds to the BMP receptors on the cell surface, resulting in the activation of downstream Smad pathways and regulating the transcription of osteogenic genes.^23^, ^45^ DEX exerts its osteoactivity via binding to the intracellular glucocorticoid receptors, which activates transcription through glucocorticoid receptor–responsive elements located in the promoters of glucocorticoid receptor target genes.^24^ It has been reported that DEX can enhance the effect of BMP‐2 on cell differentiation.^26^ The binding of BMP‐2 to its receptor activates Janus kinases (JAKs). Activated JAKs phosphorylate the signal transducer and activator of transcription 3 (STAT3). Activated STAT3 enters the nucleus and regulates transcription of multiple genes that regulate cell proliferation and osteoblast differentiation, leading to the enhancement of ALP activity. BMP‐2 and DEX synergistically increase ALP levels due to DEX‐bound GR binding to STAT3, which is recruited to the SRE by BMP‐2. Subsequently, DEX‐bound GR acts as a co‐activator for STAT3. Besides, DEX may possibly enhance the BMP‐2–regulated STAT3 transcriptional activity stimulating ALP expression by inhibiting the suppressive effect of Erk on STAT3 activity. These responses eventually result in the synergistic effects of BMP‐2 and DEX on ALP levels.^26^ Hence, the dual‐bioactive molecule–loaded fibrous meshes displayed the maximum staining intensity of alizarin red and the highest expression level of osteogenic genes. It also indicated that the core‐shell structure of electrospun fibres maintained the activity of BMP‐2 and DEX. The results of in vivo osteogenesis revealed a similar trend. Through reserving and sustainably releasing of BMP‐2 and DEX, PLA/PEG‐DEX‐BMP‐2 core‐shell fibrous meshes exhibited a maximum capacity to repair bone defect.

## 5 CONCLUSIONS

A novel core‐shell fibrous mesh loaded with DEX and BMP‐2 was fabricated through coaxial electrospinning to enhance bone regeneration. Through embedding BMP‐2 and DEX‐loaded micelles into the core of fibres, a relatively long sustainable release of both bioactive molecules was obtained. Based on the synergistic effect of BMP‐2 and DEX, an enhanced bone regeneration can be achieved. This core‐shell fibrous mesh loaded with DEX and BMP‐2 stands as a strong potential candidate for repairing bone defects.

## CONFLICT OF INTERESTS

There are no conflicts to declare.

## AUTHOR CONTRIBUTIONS

Xian Liu, Chongyun Bao and Shaobing Zhou contributed substantially to the conception and design of the experiments. Zhenzhen Wu conducted all experiments and wrote the manuscript. Tao Yang and Long Li were responsible for the synthesis and characterization of materials. Mingzheng Li and Xiaodong Guo conducted the cell experiment. Liao Wang and En Luo were responsible for the in vivo study. Yingjie Yu and Yushu Wang conducted data analyses and modified the draft.

## Data Availability

The data used to support the findings of this study are available from the corresponding author upon request.
